# New insight into the molecular control of bacterial functional amyloids

**DOI:** 10.3389/fcimb.2015.00033

**Published:** 2015-04-08

**Authors:** Jonathan D. Taylor, Steve J. Matthews

**Affiliations:** Department of Life Sciences, Faculty of Natural Sciences, Imperial College of Science, Technology and MedicineLondon, UK

**Keywords:** amyloid, chaperone, secretion, curli, biofilm, Alzheimer's, Parkinson's

## Abstract

Amyloid protein structure has been discovered in a variety of functional or pathogenic contexts. What distinguishes the former from the latter is that functional amyloid systems possess dedicated molecular control systems that determine the timing, location, and structure of the fibers. Failure to guide this process can result in cytotoxicity, as observed in several pathologies like Alzheimer's and Parkinson's Disease. Many gram-negative bacteria produce an extracellular amyloid fiber known as curli via a multi-component secretion system. During this process, aggregation-prone, semi-folded curli subunits have to cross the periplasm and outer-membrane and self-assemble into surface-attached fibers. Two recent breakthroughs have provided molecular details regarding periplasmic chaperoning and subunit secretion. This review offers a combined perspective on these first mechanistic insights into the curli system.

## Introduction

In contrast to disease-associated amyloids that underlie neurodegenerative disorders like Alzheimer's and Parkinson's disease, organisms often exploit amyloid structures for their unique mechanical and biological properties. Many bacterial species are programmed to produce extracellular amyloid protein fibers as part of a switch to a biofilm lifestyle, as such fibers offer protection from environmental stresses as well as mediate adherence to both biotic and abiotic surfaces (Larsen et al., 2007; Otzen and Nielsen, 2008; Jordal et al., 2009; Dueholm et al., 2010; Romero et al., 2010; Blanco et al., 2012; Garcia et al., 2013). Such amyloid systems are found within both gram-positive and gram-negative bacteria and appear unrelated to each other in terms of components and complexity.

The exquisite ability of bacteria to handle the production, secretion and aggregation of an otherwise toxic protein suggests there are lessons to be learned in understanding other occurrences of amyloid structure—both functional and disease-associated. The best-understood bacterial amyloid system is curli from *E. coli*, which was first reported 25 years ago as a novel, coiled proteinaceous fiber that bound fibronectin (Olsen et al., 1989). Many fascinating features have been subsequently uncovered, most notably the utility of amyloid structure (Chapman et al., 2002; Shewmaker et al., 2009), complexity of genetic regulation (Zakikhany et al., 2010; Mika and Hengge, 2013; Soo and Wood, 2013), dedicated export machinery (Robinson et al., 2006; Epstein et al., 2009), host responses to curli (Tukel et al., 2009; Rapsinski et al., 2015) and its potential in biotechnology (White et al., 1999; Sivanathan and Hochschild, 2013; Nguyen et al., 2014; Van Gerven et al., 2014).

Two recent ground-breaking studies have illuminated the underlying molecular mechanisms and help answer some fundamental questions that have puzzled the functional amyloid field for years (Cao et al., 2014; Goyal et al., 2014; Evans et al., 2015). Perhaps the most pertinent is how do organisms access the favorable properties of amyloid-like structures and yet avoid the associated cytotoxicity? Of particular interest is the means by which bacteria can inhibit amyloidogenesis during transport within the cell and then export aggregative amyloid subunits efficiently.

## How are curli subunits secreted across the outer membrane?

**Figure 1:**
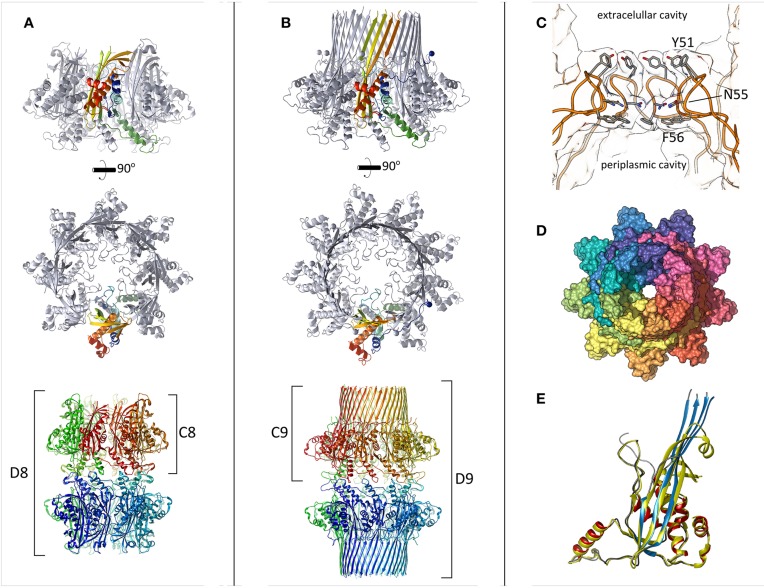
**The structure of CsgG. (A)** CsgG C16S mutant crystallized as an octamer with the transmembrane region buried or disordered. One protomer is colored in a gradient (N-terminus = blue to C-terminus = red). The lower panel shows the crystallographic symmetry encountered. **(B)** Wild type CsgG forms nonamers. The N-terminal lipidation sites are marked by spheres. Coloring as in **(A)**. **(C)** The central pore loops across which curli subunits pass is displayed by slicing through the center of the pore. Highly conserved side-chains are shown as sticks. **(D)** Surface representation of wild type CsgG, viewed from the exterior of the cell. The N-terminal ~20 residues wrap around the adjacent protomer. **(E)** Structural alignment between CsgG monomers from the “pre-pore” state (yellow) and membrane-inserted state (red helices, blue strands, gray loops). The RMSD is 0.85 Å.

The central component of the curli system, CsgG, acts as the membrane channel for secretion (Loferer et al., 1997; Robinson et al., 2006). Despite early reports of CsgA-related specificity, in isolation CsgG allows both inward and outward flux of macromolecules below a certain size and can thus be considered an ungated nanopore (Robinson et al., 2006; Taylor et al., 2011). Due to the absence of ATP within the periplasm and lack of chemical gradients, the energy source for efficient secretion of curli subunits is not known. CsgG is targeted to the outer membrane by the Lol transport system during which it is lipidated at its mature, N-terminal cysteine prior to outer-membrane insertion (Loferer et al., 1997). Although mutation of the cysteine prevents insertion of CsgG into the outer-membrane, it is able to fold independently within the periplasm (Goyal et al., 2013, 2014). This “soluble” species could be purified in the absence of detergent and its crystallization resulted in a high resolution structure of CsgG, solved using standard seleno-methionine phasing methods (Figure 1A). In this state, CsgG crystallized as two ring-shaped octamers arranged in D8 symmetry, interacting in a tail-to-tail fashion, with no transmembrane strands or helices apparent. Remaut and colleagues referred to the CsgG C8 configuration as a “pre-pore” state since it resembles a class of pore-forming proteins (PFPs) that oligomerise prior to membrane insertion (Iacovache et al., 2010).

The atomic structure of full-length, lipidated CsgG, extracted from the outer-membrane, was subsequently obtained by the same group using an ingenious approach. Inspection of crystal symmetry revealed a surprising, double-nonameric form in D9 symmetry, which was supported by negative-stain electron micrographs (Goyal et al., 2014). Despite the structural similarity between CsgG monomers in the octameric vs. nonameric form (Figure 1E), Remaut and colleagues failed to solve the structure of the nonamer by molecular replacement using coordinates from the octameric species as a search model. Instead, reasoning that intermolecular contacts would be preserved they created a bespoke C9 nonameric search model by repositioning the subunits accordingly. This allowed calculation of interpretable electron density maps and manual building of the transmembrane beta-strands to yield the first full structure of CsgG (Figures 1B–D).

CsgG clearly displays some flexibility in its oligomeric status, as has been observed for other membrane proteins, including PFPs (Iacovache et al., 2010; Gandhi et al., 2011; Yamashita et al., 2011). From analogy to PFPs, we anticipate that freshly-synthesized CsgG will oligomerise prior to membrane insertion, and thus the “pre-pore” structure gives an indication of CsgG topology prior to insertion. The different stoichiometries observed for detergent-solubilised and “pre-pore” CsgG are likely influenced by experimental conditions (e.g., concentration and detergent), as Goyal et al. themselves note. Measurements of membrane-embedded stoichiometry are thus required to reveal the physiological form of CsgG. Given the plasticity PFP stoichiometry and the widespread presence of CsgG across bacterial phyla, we anticipate that CsgG homologs will form a variety of oligomeric states.

The crystal structure of a proteolytically digested form of membrane-bound CsgG, solved using experimental phasing methods was subsequently reported by another group (Cao et al., 2014). Both nonamers (C9) and double-nonamers (D9) could be purified and crystallized, however unlike the full-length structure, only the C9 form produced crystals that diffracted sufficiently. Mass spectrometry analysis showed that chymotrypsin removes the first 34 and last 12 residues of the mature protein. Thus, in this case, lipidation was required for proper insertion and extraction from the membrane but not for stability within detergent micelles (Robinson et al., 2006).

**Figure 2:**
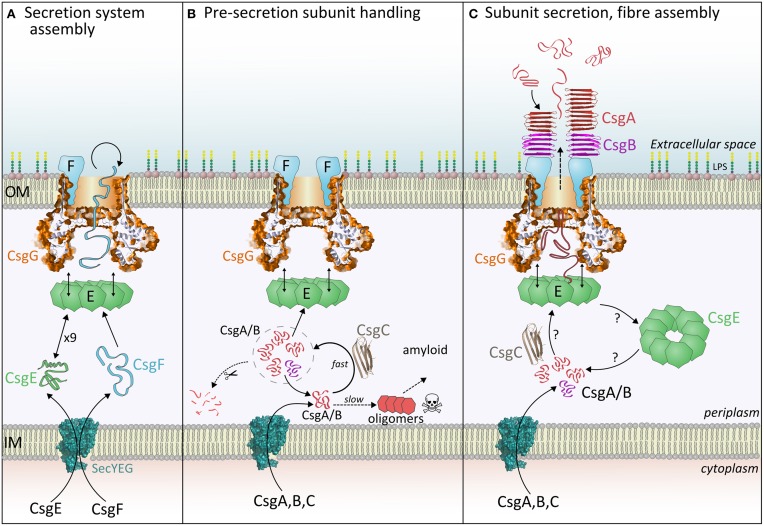
**The three stages of curli fiber biogenesis. (A)** Secretion system assembly: CsgE and CsgG achieve the translocation of CsgF, which folds and binds CsgG. Loss of CsgE results in a dramatic reduction in surface display of CsgF. **(B)** Pre-secretion subunit handling: Incoming CsgA or CsgB monomers interact with CsgC, which delays spontaneous formation of toxic oligomers. It is expected that subunits within this CsgC-buffered pool (signified by the dashed circle) are either secreted, digested or revert to an amyloidogenic pathway. **(C)** Subunit secretion, nucleation and fiber assembly: Each curli subunit encounters CsgE (nonamers?) and becomes trapped within the periplasmic cavity of CsgG. Partially folded subunits then traverse the central pore and are released into the extracellular milieu. The folding pathway of periplasmic CsgA determines the appearance and properties of extracellular fibers, thus it is unlikely that CsgA is secreted as a linear polypeptide. Once outside the cell, CsgB is interacts with CsgF and initiates nucleation of the CsgA fiber.

CsgG occurs in the outer-membrane as a novel nonameric, 36-stranded beta-barrel, with a central pore allowing passage for outgoing curli subunits (Figure 2). A remarkable feature of this protein is the large cavity (5 nm) within the β-barrel, which in other large membrane proteins is typically filled by a plug domain (e.g., FhuA, Ferguson et al., 1998 and PapC, Remaut et al., 2008). The wall of this cavity is lined with conserved polar or negatively-charged side-chains, which are thought to be important for proper export of curli subunits (Cao et al., 2014). CsgF is surface-localized and anchors CsgB at the base of nascent fibers (Nenninger et al., 2009). It is assumed that CsgG is responsible for secretion of CsgF however this has not been directly proven. Given the unusual absence of large extracellular loops within CsgG, it seems conceivable that CsgF inserts into this cavity and presents a binding site for CsgB (Figure 2). Although Cao et al. tested point mutants to probe the importance of this region, only a triple mutant showed a significant defect in curli formation (Cao et al., 2014).

At the interface between the periplasmic domain and the outer-membrane, a highly conserved loop region constricts the pore to 1.2 nm, which could accommodate some small folded elements of secondary structure within substrates (Figures 1C–D). Previous measurements from electron microscopy suggested a pore diameter of 2 nm (Robinson et al., 2006). The constriction loop observed in CsgG is similar in position and hydrophobic character to that of the Anthrax PA_63_ toxin (Krantz et al., 2005). In that system, researchers found that widening the pore or making it more hydrophilic actually reduced the overall rate of substrate translocation. It was proposed that the narrow pore lined with hydrophobic and aromatic residues would reduce the energetic cost of unfolding, and allow the substrate to be ratcheted through (Krantz et al., 2005). Cation-π interactions may also play a role in this process. Thus, the central constriction controls both the maximum diameter and the identity of substrate proteins by selecting only those that can unfold sufficiently. Other protein secretion systems also feature hydrophobic pinch points, such as the Sec61 or SecY complex (Van Den Berg et al., 2004).

What do the structures of CsgG tell us about the mechanism of subunit translocation? Given the lack of ATP or chemical gradients across the outer-membrane it seems likely that translocation of curli subunits and CsgF will be driven by the energetics of amyloid subunit folding and sequential transient interactions. Recent evidence suggests that CsgE may play an important role in this process. CsgE recognizes native and foreign CsgA-like substrates if they possess the N-terminal 22 residue peptide or a curli repeat sequence (Robinson et al., 2006). Goyal et al. showed that CsgE forms a mixture of nonamers and monomers in solution, and the nonamer forms a cap at the base of the CsgG periplasmic domain, leading to a blockade of electrical conductivity through isolated pores (Goyal et al., 2014). A mechanism was proposed whereby each curli subunit is introduced by CsgE into the periplasmic vestibule of CsgG, and subsequently becomes trapped (Figure 2C). This is reminiscent of the GroEL/GroES chaperone system thus, by analogy, captured polypeptides achieve the correct (un)folded state to interact with and pass through the central loops of CsgG. The whole process descends an energy gradient determined by favorable interactions and increasing entropy.

Thus, CsgE is thought to select potential substrates and maximize their secretion efficiency by playing a vital role in subunit export. This is entirely consistent with the behavior of *csgE*^−^ strains which accept foreign substrates and form drastically fewer fibers, themselves morphologically distinct to wild type fibers (Gibson et al., 2007; Nenninger et al., 2011). Thus CsgG possesses inherent polypeptide secretion activity in the absence of CsgE. Indeed it is possible to purify CsgA directly from the supernatant simply by co-expressing it with CsgG (Zhou et al., 2013). A recent survey of curli operons across all bacterial kingdoms showed that many species within the Alphaproteobacteria clade lack a CsgE homolog (Dueholm et al., 2012). Also, there is diversity in the size of curli subunits, with some bacteria synthesizing CsgA-like subunits that are 3–4 times larger than in *E. coli*. It does not seem likely that these larger secretion substrates can be fully entrapped within the CsgE-CsgG complex, unless those bacteria can create a larger cavity (e.g., by forming oligomers larger than a nonamer). Moreover, attempts to translocate CsgA-fusion proteins of various lengths and folding states show that much longer polypeptides (up to 8 times) are acceptable so long as their structure remains dynamic prior to export (Chen et al., 2014; Van Gerven et al., 2014). Perhaps substrates are not fully entrapped during the early stages of secretion and are instead fed into the cavity by CsgE and the CsgE-CsgG complex closes as the subunit is secreted.

The exact sequence of interactions between CsgA, CsgE, and CsgG remains unknown (Figure 2C). Goyal et al. presented three potential routes consistent with our current understanding (Goyal et al., 2014). Future studies will determine if CsgE monomers or nonamers are responsible for sequestering individual curli subunits, and probe the dynamic interaction between CsgE and CsgG. Furthermore, the region of the substrate that enters the CsgG pore first is not known, however the role of the N-terminal, gly/gln-rich region in targeting CsgA to CsgE suggests that this section of the polypeptide is the last to exit the pore.

The lack of 2-fold symmetry within nonameric CsgG encourages the question of how many fibers each secretion system can produce. As yet, this has not been adequately addressed, however the formation of nonameric CsgE/G complexes hints that CsgF may also interact with CsgG as a symmetric ring. Since CsgF is responsible for localisation of CsgB this could allow the creation of up to 9 curli fibers. Future studies should focus on determining the full stoichiometry of the secretion system.

Finally, there is reason to suspect that the structure of isolated CsgG that we now have is different from its physiological counterpart—the CsgEFG co-complex. Epstein et al. demonstrated that CsgG has varying thermal- and SDS-sensitivity when extracted from various *csg* deletion mutants (Epstein et al., 2009). Thus as the individual proteins within the secretion system affect the stability of CsgG, they likely also affect its ultrastructure.

## How is premature amyloidogenesis controlled?

Consideration of the export mechanism leads naturally to the question: What happens to curli subunits within the periplasm prior to secretion? It is this question that is addressed by the other recent discovery regarding the most enigmatic curli protein, CsgC (Evans et al., 2015). Newly synthesized, monomeric subunits within the periplasm pose a problem for the cell, since formation of toxic pre-amyloid species disrupts the proteostatic balance (Cheng et al., 2013; Evans et al., 2015). In the absence of CsgG, curli subunits are rapidly digested in the periplasm before they can cause toxicity (Loferer et al., 1997). The Chapman group hypothesized that some factor(s) must inhibit premature amyloidogenesis thereby allowing continued access for proteases. Through careful examination of periplasmic extracts it was shown that the curli protein CsgC is responsible for this phenomenon. Cells lacking CsgG and CsgC accumulate intracellular amyloid, and *in vitro* CsgC directly inhibits CsgA fibrillation at impressive substoichiometric molar ratios. Previous studies have reported the inhibitory effect of CsgE and other bacterial chaperones on amyloid formation by CsgA however CsgC is a far more efficient inhibitor of this process (Evans et al., 2011; Nenninger et al., 2011).

For many years the role of CsgC had remained elusive. In Gammaproteobacteria, *csgC* is the only other gene co-transcribed with the curli subunits, which hints at CsgC having a role in their cellular fate. However, the low abundance of CsgC within the periplasm argues against formation of 1:1 complexes with subunits, as occurs within the bacterial chaperone-usher system (Gibson et al., 2007). The mechanism by which bacteria restrict transcription of *csgC* remains unknown, however, the discovery that each molecule of CsgC can effectively inhibit amyloid formation amongst a pool of several 100 CsgAs over the course of many hours is consistent with its extremely low abundance. These molar ratios imply that CsgC is able to influence the nucleation and/or elongation rate of CsgA, perhaps by diverting its folding pathway or acting on oligomers or nascent fibril ends. A rigorous kinetic analysis of fibrillation would reveal the likely inhibitory mechanism, as has been recently demonstrated for other amyloid systems (Cohen et al., 2015; Galvagnion et al., 2015). The molecular mechanisms governing this transient and dynamic interaction are a source of great interest since, curiously, CsgC can also inhibit formation of amyloid by α-synuclein, but not Aβ42 peptide.

An interesting question to explore is whether indeed the *primary* function of CsgC is actually to inhibit premature amyloid formation. Biophysical measurements show that freshly-purified CsgA remains fairly unstructured and non-aggregated in the presence of CsgC over many hours (Evans et al., 2015). Depending on the molar ratio, CsgA is eventually able to escape this containment and form Thioflavin T-binding amyloid. It is not known if these fibers are structured differently to those formed by CsgA alone, however a knockout of *csgC* produces polymorphic extracellular fibers (Gibson et al., 2007). Thus the primary purpose of CsgC may be to divert the pool of CsgA monomers into a specific structural state suitable for translocation and nucleation and this inherently results in the inhibition amyloid formation. Polymorphism in fiber structure is a frequently encountered with amyloidogenic proteins and is dependent on fibrillation conditions. CsgC may prevent this inefficiency by pre-disposing CsgA to fold and nucleate in a particular manner. CsgC can thus be thought of as being akin to a chaperone or holdase in function (Figure 2B). Much effort has gone into identifying small-molecules, peptides or immunoglobulin-based inhibitors of amyloidogenesis. CsgC (whose structure resembles an immunoglobulin fold) is one of the most potent examples known and a molecular understanding of this capability would empower development of medically-useful analogs. Furthermore, we propose that other functional amyloid systems may possess their own specific inhibitor to avoid premature fibrillation.

The structure of CsgC is known and it was originally proposed that it may influence the porosity of CsgG via a redox mechanism employing its CxC motif (Taylor et al., 2011). The recent structure of CsgG is not consistent with this hypothesis. The free cysteine residue in CsgG is buried and regardless, pair-wise disulphide bonding is unlikely in an oligomer containing an odd number of protomers. The role of the CxC motif within CsgC remains mysterious—it is required for stability within the periplasm, but not for inhibitory function *in vitro* (unpublished data). The fact that CsgC interacts with CsgA and affects its folding pathway makes much sense of the previous data, however there remains one unexplained observation: Wild type cells are resistant to bile salt influx, however they become sensitive upon deletion of the *csgBA* genes (see Figure 6 in Taylor et al., 2011). In this state the CsgEFG pore complex would have no subunits to export and bile salts can therefore diffuse into the cell via the CsgG pore. Curiously, when the *csgBAC* genes are deleted bile salt resistance returns, suggesting a difference in the CsgEFG complex. One explanation is that CsgC plays a role in structural preparation of CsgF for export and in its absence CsgF blocks the pore. CsgF is somewhat amyloidogenic and is as rich in Asn/Gln residues as curli subunits. Alternatively CsgC may interact with CsgE to promote release from CsgG or a conformation change that renders the pore accessible. These intriguing results suggest that CsgC may play a wider role in curli biogenesis than just inhibition of amyloidogenesis by CsgA and CsgB.

The outstanding questions within the curli field concern the structure and location of secretion components CsgE and CsgF and the mechanisms of nucleation by CsgB and fiber extension. There is growing interest in use of the curli secretion system for biotechnological purposes, for instance by decorating fibers with bespoke chemistry, creating nanowires, and as adhesive materials (Chen et al., 2014; Nguyen et al., 2014; Van Gerven et al., 2014; Zhong et al., 2014). Whilst the major interest of synthetic biology in the curli system revolves around utilizing the self-assembling and highly stable nature of the amyloid subunits, the reported structure of CsgG enables others to explore its use as a nanopore for DNA sequencing or chip-based detection of biological interactions (Nivala et al., 2013). Future research into the complex and dynamic process of secretion will empower these efforts greatly. Furthermore, our improved understanding of how these bacterial systems operate not only provides intriguing insight into disarming amyloids in biofilms, but placing future findings in the context of human amyloids may illuminate new avenues for treating protein-folding diseases.

### Conflict of interest statement

The authors declare that the research was conducted in the absence of any commercial or financial relationships that could be construed as a potential conflict of interest.
